# Diffusion tensor imaging in cerebral small vessel disease applications: opportunities and challenges

**DOI:** 10.3389/fnins.2024.1473462

**Published:** 2024-10-16

**Authors:** Siyu Yang, Yihao Zhou, Feng Wang, Xuesong He, Xuan Cui, Shaojie Cai, Xingyan Zhu, Dongyan Wang

**Affiliations:** ^1^Department of Acupuncture and Moxibustion, Heilongjiang University of Chinese Medicine, Harbin, China; ^2^Department of CT and Magnetic Resonance, The First Hospital Affiliated to Heilongjiang University of Chinese Medicine, Harbin, China; ^3^Department of CT and Magnetic Resonance, The Second Hospital Affiliated to Heilongjiang University of Chinese Medicine, Harbin, China; ^4^Department of Peripheral Vascular, The First Hospital Affiliated to Heilongjiang University of Chinese Medicine, Harbin, China; ^5^Department of Geriatrics, The Second Affiliated Hospital of Heilongjiang University of Chinese Medicine, Harbin, China; ^6^Department of Acupuncture and Moxibustion, The Second Hospital Affiliated to Heilongjiang University of Chinese Medicine, Harbin, China

**Keywords:** DTI, CSVD, DKI, glymphatic system, preclinical imaging features

## Abstract

Cerebral small vessel disease (CSVD) is a syndrome of pathology, imaging, and clinical manifestations caused primarily by a variety of functional or structural lesions in the small blood vessels of the brain. CSVD contributes to approximately 45% of dementia and 25% of ischemic strokes worldwide and is one of the most important causes of disability. The disease progresses insidiously, and patients often have no typical symptoms in the early stages, but have an increased risk of stroke, death, and poor long-term prognosis. Therefore, early diagnosis of CSVD is particularly important. Neuroimaging is the most important diagnostic tool used for CSVD. Therefore, it is important to explore the imaging mechanisms of CSVD for its early diagnosis and precise treatment. In this article, we review the principles and analysis methods of DTI, analyze the latest DTI studies on CSVD, clarify the disease-lesion mapping relationships between cerebral white matter (WM) microstructural damage and CSVD, explore the pathogenic mechanisms and preclinical imaging features of CSVD, and summarize the latest research directions of CSVD and research methods to provide a comprehensive and objective imaging basis for the diagnosis and treatment of CSVD.

## 1 Introduction

Cerebral small vessel disease (CSVD) is a syndrome of pathology, imaging, and clinical manifestations caused primarily by various functional or structural lesions of the small blood vessels of the brain (Wardlaw et al., 2019). CSVD contributes to approximately 45% of dementia and 25% of ischemic strokes worldwide and is one of the most important causes of disability (Pasi et al., 2016). CSVD is a highly age-related chronic disease and is considered a major vascular factor in stroke, dementia, cognitive decline, gait impairment, and mood disorders (Ter Telgte et al., 2018). The detection rate of asymptomatic cerebral infarction in healthy older adults is as high as 20–50%, most of which are lacunar infarcts (LI) (Dey et al., 2016). The disease progresses insidiously, and patients often have no typical symptoms in the early stages. However, the risk of stroke and death is increased, and the long-term prognosis is poor. Vascular factors may act synergistically with pathological changes in neurodegeneration to increase the risk of cognitive decline and dementia (Zhang et al., 2017). Despite the enormous global burden of CSVD in causing stroke, dementia, and disability, there are few effective treatments for this disease (Smith and Markus, 2020). Therefore, it is important to explore the pathogenesis and diagnostic markers of CSVD.

Neuroimaging is the most important modality for the diagnosis of CSVD. According to the International Neuroimaging Standards, the total CSVD score consists of four components: lacunar infarcts, white matter hyperintensities (WMH), cerebral microbleeds (CMBs), and enlarged perivascular spaces (PVS) (Duering et al., 2023). These CSVD markers are usually observed on brain imaging in normal elderly subjects, with a prevalence of 8–28% for lacunae, 5–23% for CMBs, and 50–98% for WMH (Hilal et al., 2017). Diffusion tensor imaging (DTI) can infer the structural features of the brain based on the diffusion properties of water molecules and assess the integrity of the WM tracts (Pasquini et al., 2022). The main markers used by DTI to assess WM damage include fractional anisotropy (FA), mean diffusivity (MD), etc. The main DTI analysis methods include ROI-based analysis, white matter tract-based analysis, voxel-based analysis, and brain structure network-based analysis. Innovations in the DTI analysis method have made it more accurate for the assessment of WM injury while providing a more effective method for the assessment of patients with CSVD (Finsterwalder et al., 2020). Therefore, the application of DTI to explore the imaging mechanism of CSVD is important for its early diagnosis and precise treatment. This article reviews the principles and analytical methods of DTI, describes the limitations and improvement methods of the DTI technique, analyzes the latest DTI studies on CSVD, clarifies the disease-lesion mapping relationships between WM microstructural damage and CSVD, explores the pathogenic mechanisms and preclinical imaging features of CSVD, and summarizes the latest research directions of CSVD. This review aims to provide a comprehensive and objective imaging basis for the diagnosis and treatment of CSVD.

## 2 DTI principles

DTI was first proposed by Basser et al. (1994) and was developed and optimized using diffusion-weighted imaging(DWI). Traditional DWI is an imaging technique that is based on the Brownian motion of water molecules. However, because of the barrier of anisotropic diffusion in biological tissues, Gaussian motion does not match the actual motion of water molecules. Therefore, a second-order diffusion tensor was introduced, resulting in DTI. The emergence of DTI allows us to observe the orientation and microstructural integrity of nerve fiber bundles in three dimensions (Mascalchi et al., 2019). Both DWI and DTI are MRI techniques used to characterize the diffusion directions of water molecules. The diffusion of water molecules within a voxel in various directions is measured by varying the direction of the diffusion-sensitive gradient. DWI describes the diffusion of water molecules in one or more specific directions, whereas DTI usually involves the diffusion of water molecules in 12 to 30 directions, which provides a more accurate description of the diffusion movement of water molecules (Magdoom et al., 2023). In each voxel, DTI represents anisotropic diffusion through an ellipsoid that can be mathematically modeled using a 3 × 3 matrix (called a tensor). The model allows the representation of the diffusion of water molecules in three dimensions, describing the different directions and “intensities” of the motion (Pasquini et al., 2022). DTI is regarded as a promising alternative biomarker for monitoring the progression of WM injury in patients with CSVD because of its noninvasive nature and high sensitivity (van den Brink et al., 2023). Although DTI is significantly better than conventional MRI in determining nerve fiber myelin lesions. However, due to its limitations, it is not effective in determining the integrity of nerve myelin in brain regions with low anisotropy or a bias toward isotropy (Guo et al., 2016; Guo et al., 2016). When DTI is applied to evaluate CSVD, the advantages and disadvantages of the technique should be clarified.

## 3 DTI main analysis methods

The main DTI analysis methods include ROI-based analysis, white matter tract-based analysis, voxel-based analysis, and brain structure network-based analysis. The potential applications and advantages and disadvantages of each DTI analysis method we present in Table 1.

**Table 1:** DTI main analysis method.

Analysis method	Potential applications	Strength	Challenge
**ROI-based analysis**			
ROI-based analysis	Analysis of specific brain regions or fiber tracts	Can be split manually or by template	There are limitations to the split approach
**White matter tract-based analysis**			
DTT	Deterministic fiber tracing	Enables *in vivo* viewing and study of neural network connectivity and continuity	Does not convey information about the strength of the connection. Cannot resolve white matter fiber crossings
	Probabilistic fiber tracing	Some resolution of fiber crossover issues	Computationally intensive and affected by anatomy
AFQ	Automated extraction fiber tracts	Estimation of diffusion parameters of fiber bundles at point-by-point along the fiber trajectory	
**Voxel-based analysis**			
VBA	Whole-brain voxel levels for spatially localized diffusion studies	Comprehensiveness, objectivity and repeatability	Errors due to alignment, smoothing, etc.
TBSS	Automated analysis of diffusion tensor data	Overcoming the problem of alignment error or smoothing kernel selection reduces the occurrence of false positive results.	Arbitrary pixels between different data hardly correspond to the same fiber bundle
PSMD	Evaluation of CSVD	Elimination of cerebrospinal fluid contamination improves capture sensitivity Calculations are fully automated	
**Structural Brain Network-based Analysis**			
Graph theory	Computing Brain Network Topological Properties of the Whole Brain	Showing whole-brain information changes induced by injury to different structures	Lack of normality in the selection of nodal brain regions

### 3.1 Region of interest based analysis

ROI-based analysis is based on the study of specific brain regions or fiber tracts, which need to be corrected manually or by templates when setting the region of interest, and subsequently, the mean or median values of the indexes of the relevant voxels within the region. The analysis of changes in white matter microstructural properties in combination with pathologic findings and specific ROIs helps to explore the potential mechanisms of altered DTI-derived parameters in patients with CSVD. However, the manner in which ROIs are split into regions suffers from the discrepancy between manual selection and the inadequacy of template-based automated region splitting (Auriel et al., 2014).

### 3.2 White matter tract-based analysis

Diffusion tensor tractography (DTT) is a technique that builds on DTI data and can display the alignment of cerebral white matter bundles *in vivo*. Through this technique, we can not only visualize the morphology of WM fibers but also extract the diffusion index of the fiber bundles for quantitative statistical analysis. Currently, the two most commonly used methods for diffusion tensor fiber tracking are deterministic and probabilistic fiber tracking (O'Donnell and Westin, 2011). The deterministic fiber-tracking method starts from the seed point, and at each step, a definite fiber direction is obtained based on the diffusion direction distribution function or diffusion tensor principal direction at the current point, leaps forward according to this direction, and repeats the process until the termination condition is satisfied. Probabilistic fiber tracking methods mainly use the known information of the fibers to obtain the *a posteriori* probability distribution of the fibers. The direction at each step is sampled and selected from the probability distribution, and finally, the probabilistic path from the seed point to the target region is obtained (Behrens et al., 2007). However, each analysis method has limitations. Deterministic fiber tracing is useful for resolving complex fiber patterns, especially when performing standard diffusion tensor modelling of the data. It does not convey information about the strength of the connections (Jones, 2008). The disadvantage of probabilistic fiber tracing methods is that the calculations can be computationally intensive. The number of streamlines through each voxel has been used as a proxy for connection strength, although this is an oversimplified interpretation that may be affected by anatomically incorrect reconstructions or complex fiber arrangements (Grier et al., 2020). Automated fiber quantification (AFQ) overcomes the limitations of both (Qiu et al., 2021). The AFQ is a quantitative white matter analysis technique based on DTI sequences that automatically extracts 20 major WM tracts from the whole brain. It can precisely locate and estimate the point-by-point diffusion parameters of each specific fiber bundle at 100 anatomically equivalent locations along the fiber trajectory, providing more accurate information for quantitative analysis (Huang et al., 2020).

### 3.3 Voxel-based analysis

Voxel-based analysis (VBA) is an analytical method for spatially localized diffusion studies at whole-brain voxel levels. Compared to ROI-based methods, VBA does not require *a priori* knowledge of the researcher, is not subjective to the researcher’s influence and confounding factors, and is characterized by comprehensiveness, objectivity, and reproducibility. However, this method may be affected by the alignment, smoothing, and other factors. Therefore, when designing experiments, cluster thresholds and *p*-values are strictly set, and it is not possible to determine whether there are real differences or errors due to alignment; thus, they have been used less frequently (Raffelt et al., 2017).

Tract-Based Spatial Statistics (TBSS) is a spatial statistical method based on the white matter skeleton proposed by the Centre for Functional MRI of the Brain (FMRIB) at the University of Oxford to overcome the problems of alignment error or smooth kernel selection in the VBA method. The TBSS method can automatically and accurately analyze diffusion tensor data, and its core is the “skeletonization” data processing method, which realizes the alignment between different fiber bundles and significantly reduces the occurrence of false-positive results. The TBSS can provide a quantitative index for the study of white matter structural changes related to cognitive deficits and mood changes caused by CSVD. TBSS can provide quantitative indicators for studying changes in white matter structure associated with cognitive impairment and mood changes due to CSVD (Yao et al., 2017; Jha et al., 2015).

The peak width of skeletonized mean diffusivity (PSMD) is a new, fully automated CSVD parameter based on two processing techniques (TBSS and histogram analysis) of DTI data, reflecting the heterogeneity of voxel-based mean diffusivity (MD) values in major WM tracts (Zanon Zotin et al., 2023). This method eliminates cerebrospinal fluid contamination and improves the sensitivity to capture CSVD-related changes. Because PSMD is sensitive to age-related cognitive changes and calculations are fully automated, it has clear advantages in large-sample trials (Lam et al., 2019; Deary et al., 2019; Egle et al., 2022). Longitudinal analyzes showed that the PSMD had the smallest sample size estimate compared to the whole-brain mean peak diffusivity height, standardized WMH volume, brain parenchyma score, processing speed score, and standardized lacunar volume (Wei et al., 2019). Therefore, the PSMD may be of great practical value in clinical research and applications. Multiple cohort studies have shown that PSMD can be a valid assessment of cognitive impairment in cerebral small vessel disease (Zanon Zotin et al., 2023; Wei et al., 2019; Zanon Zotin et al., 2022).

### 3.4 Brain structure network-based analysis

The brain network-based analysis method uses specific brain regions as nodes and nodes’ structural connections as edges to construct a structural network and uses graph theory to calculate the corresponding topological properties of the brain network. The brain structural network is a mapping of brain structural connections, and compared with the traditional MRI markers that focus on localized brain damage, network construction focuses on whole-brain integration of different structural damage information (Griffa and Van den Heuvel, 2018). Structural brain networks represent the integrity of WM connectivity and thus arguably reflect the mechanisms of cognitive dysfunction better than any other measure (Du et al., 2019). Several cross-sectional studies of patients with CSVD have shown that reduced structural network integrity, manifested as a decrease in overall efficiency, is associated with increased cognitive impairment (Lawrence et al., 2014; Reijmer et al., 2015; Tuladhar et al., 2015) as well as an increased risk of developing dementia in the future (Tuladhar et al., 2016).

## 4 Exploring the pathogenesis of CSVD neuroimaging

Available evidence suggests that the pathogenesis of CSVD is associated with factors that impair WM tract connectivity (Mascalchi et al., 2019; Huang et al., 2020; Zhang et al., 2022). Different functions of frontal WM tract injury can lead to different types of brain connectivity disruptions. Damage to association fibers interrupts the transmission of information between cortical areas that mediate different behavioral domains. Damage to commissural fibers will lead to a disrupts the transmission of information between cerebral hemispheres. Damage to projection fibers disrupts the influence of subcortical structures on behavior. Specific segments of these white matter bundles are associated with specific brain functions such as cognition (Mascalchi et al., 2019), emotion (Li et al., 2020), and gait (van der Holst et al., 2018).

### 4.1 WM tract damage and CSVD

Alterations in WM microstructure are thought to be the main pathogenesis of CSVD (Finsterwalder et al., 2020). It is widely accepted that WM tracts in the corpus callosum contain united fibers connecting the frontal lobe (FL) to other cortical areas that may be damaged in the early stages of CSVD (Qiu et al., 2021). Two recent imaging studies (Mascalchi et al., 2019; Zhang et al., 2022) comparing the two types of mild cognitive impairment (MCI) have been conducted. It has been shown that vascular MCI and CSVD-MCI impairments are associated with microstructural changes in multiple WM tracts, mainly located in the cerebral hemispheres WM potential connections. These include the corpus callosum, which links interhemispheric information; the WM within the thalamus, which transmits thalamocortical information; and the WM in the dentate nucleus-thalamus or dentate nucleus-red nucleus-thalamus, which governs cerebellar-cerebral connections (Mascalchi et al., 2019). However, a recent study has shown that MD values in the forceps minor (Fmi) are the better indicators for differentiating between amnestic MCI (a-MCI) and CSVD-MCI. Studies have also shown that the impaired tracts in a-MCI are mainly related to memory function, whereas the impaired tracts in CSVD-MCI are mainly related to executive function (Zhang et al., 2022).

Gait disturbance, another major manifestation of CSVD, has a significant impact on patients’ lives. A study using a probabilistic fiber bundle approach suggested that the microstructural integrity of the terminal right cingulate gyrus plays an important role in gait speed in patients with MCI (Haddad et al., 2023). However, a recent Radboud University Nijmegen Diffusion tensor and Magnetic resonance imaging Cohort (RAN DMC) study found that stride length is a more sensitive marker of gait abnormality in CSVD than stride frequency and gait speed, and a 5-year follow-up study of patients with CSVD demonstrated that an increase in MD values of the corpus callosum and corona radiata area is significantly associated with a decrease in stride length in patients with CSVD (van der Holst et al., 2018).

The current study demonstrated that CSVD damage to WM tracts exhibits broad interhemispheric symmetry and is limited to specific segments. An AFQ study suggested that different WM bundle segments of the anterior thalamic radiation (ATR) may be associated with different cognitive domains. The MD values of the left ATR nodes 1–56 (anterior) were independent predictors of situational memory scores. FA values of the left ATR nodes 8–32 (anterior part) were independently correlated with verbal function, and FA values of the right ATR nodes 43–61 (middle part) were independently correlated with executive and gait function (Huang et al., 2020). To explore more precise disease-lesion mapping studies, Xie et al. (2022) conducted a meta-analysis showing that damaged regional WM detected by DTI is associated with domain-specific cognitive deficits in CSVD. The frontal lobe (FL) is closely associated with general cognition, executive function, and attention. The corpus callosum (CC) is strongly associated with memory and attention. The cingulate gyrus (CG) is closely associated with general cognition and attention. The radial crown, internal capsule, and thalamic radiation are also strongly associated with general cognition.

### 4.2 Brain network damage and CSVD

Currently, an alternative explanation for the mechanism of CSVD is considered to be a global rather than a focal disease. This is because a variety of structural damages can remotely affect structural and functional network connections (Ter Telgte et al., 2018). Previous studies have demonstrated that structural brain networks represent the integrity of WM connectivity and have advantages over traditional imaging markers in explaining cognitive dysfunction (Du et al., 2019). The rich-club is a common property of complex networks, which is essential for efficient global information transfer and complex neural functions in the brain (Riedel et al., 2022). Rich clubs work to enhance the brain’s ability to recover by maintaining the integrity of the network and promoting compensatory mechanisms when parts of the network are damaged. Tuladhar et al. (2017) showed that rich-club connection strength mediated the association of WMH with processing speed and executive function, so that higher rich-club connection strength was associated with better cognitive performance. However, a longitudinal study suggests otherwise (van Leijsen et al., 2019). The results showed that the effects of WMH on dementia were causally mediated by global network efficiency and peripheral connectivity strength, suggesting that whole-brain networks, rather than rich-club disruption, play an important role in causing cognitive decline and dementia in older adults with CSVD. A preclinical study of the progression of CSVD supports this view (Du et al., 2021).

The latest preclinical phase study of brain structural networks in the CSVD explains the above observations, with preclinical cognitive impairment (PCI) showing a weak loss of node strength but no significant abnormality in the rich-club. While patients with MCI show disruption in the rich-club, there is a more severe loss of node strength (in preference to hub nodes), as well as an overall disruption of local connectivity. This impaired topology may also underlie cognitive deficits in CSVD, particularly in the domains of attention, executive and memory cognition (Du et al., 2020). Indeed, as studies of other disorders have shown, the disruption of the rich-club reflects a decline in global communication and altered functional brain dynamics, and the two are not antagonistic. However, because the rich-club is a common property of complex networks that are less sensitive than changes in node strength versus changes in the global network, some of the studies have come to different conclusions (Shu et al., 2018).

### 4.3 Body fluid environment alterations and CSVD

Indeed, WM tract damage may be a downstream manifestation of alterations in the body fluid environment, which would be beneficial for the treatment and prevention of disease. Previous studies have shown that Type 2 diabetes mellitus (T2DM) can lead to WMH (Teng et al., 2022), lacunar infarcts (Zhou et al., 2022) and CMBs (Chen et al., 2021) and is strongly associated with total CSVD load. Liu et al. (2024) found that the mechanism of visuospatial function decline in T2DM patients was associated with deterioration of the right inferior longitudinal fasciculus (ILF), and that impairment of callosum forceps minor (CF_minor) and the right inferior fronto-occipital fasciculus (IFOF) was closely associated with increased CSVD burden. Another study showed that fibrin-like degeneration of perforating arteries, capillaries, and small veins is another pathogenesis of CSVD (De Silva and Faraci, 2020). Studies have shown that lower lenticulostriate artery (LSA) counts are associated with the development of diabetes mellitus, larger DWMH volume ratios, and higher degrees of fiber bundle damage in most brain regions. As the most common brain-penetrating artery, the LSA supplies blood to the basal ganglia and internal capsule regions, where 35–44% of ischemic and hemorrhagic strokes occur (Xie et al., 2021), and loss of the LSA may be associated with more severe damage to the microstructure of the WM (Zhang et al., 2023).

To further understand the association between risk factors and microstructural changes, a new pathway for circulation and waste removal from cerebrospinal fluid (CSF), the glymphatic system (GS), has been proposed. In the GS, CSF flows into the periarterial space for exchange with the interstitium within the brain parenchyma and out of the perivascular space for the removal of metabolic wastes and solutions (Jiang, 2019). Impaired GS clearance leads to excessive accumulation of cellular debris and metabolic waste in the perivascular space, which in turn exacerbates decreased cerebrovascular reactivity, blood–brain barrier disruption, and perivascular inflammation. Ultimately, cognitive impairment is the primary physiological consequence of lymphatic system failure (Taoka et al., 2017). Previous studies have calculated clearance in rodent brains using intrathecally injected gadolinium contrast and fluorescent tracer methods (Cai et al., 2021; Christensen et al., 2020). However, the tracer-based methods used to assess GS alterations are invasive and unsuitable for human studies. A new noninvasive DTI algorithm assesses GS activity by calculating the diffusion coefficient in the perivascular space DTI-ALPS (Taoka et al., 2017).

A community-based study demonstrated that (Tian et al., 2023) Glymphatic injuries assessed by low DTI-ALPS indices were associated with the presence, severity, and specific neuroimaging phenotypes of CSVD in a community-based population. It is worth mentioning that the study included a total of 2,219 subjects, which is the largest known study on DTI-ALPS. In a cross-sectional study of 133 patients with CSVD, the DTI-ALPS index was highly correlated with a comprehensive neuropsychological test in the area of cognitive impairment (Tang et al., 2022). A related study also reported that a low DTI-ALPS index mediates the association between WMH and situational memory in patients with CSVD (Ke et al., 2022). When the GS is weakened or dysfunctional, CSF-interstitial fluid exchange and drainage are impeded, and accumulation of cerebral metabolites (including toxins and Aβ proteins) leads to a neuroinflammatory response and a further increase in the volume of interstitial fluids in the brain tissue (Zhang et al., 2021; Thrippleton et al., 2019; Zhang et al., 2022). Recent studies suggest that (Lan et al., 2022) increased extracellular fluid volume may occur first, followed by demyelination and axonal damage in WM. Thus, microstructural changes in patients with cerebral small vessel disease may be mediated by extracellular fluid accumulation, and an increased extracellular fluid volume in the WM is associated with decreased FA. In addition, further imaging studies have shown that changes in extracellular fluid volume, as assessed by the DTI index of free water (FW) in the WM, correlate with CSVD-MR markers (PWMH, DWMH, and PVS) and CSVD burden. Thus, extracellular fluid volume in WM is thought to play an important role in the severity of cerebral small vessel disease (Lan et al., 2022).

## 5 CSVD preclinical stage identification

The most important significance of CSVD neuroimaging studies is the early diagnosis of the disease through imaging markers; however, traditional imaging markers such as WMH, lacunes, PSV, CMBs, recent small subcortical infarcts, and brain atrophy are irreversible once they occur. Localization and diagnosis of CSVD in the preclinical stage, as well as before the occurrence of traditional imaging markers, has become a recent research hotspot. There is growing evidence that some tissue changes also occur around the WMH, and these specific, subtle changes in the normal appearance of the white matter (NAWM) are referred to as “white matter high signal penumbra (WMH-P)” (Wu et al., 2019). Recognizing and assessing these specific and subtle changes in the penumbra may influence early treatment decisions. It may be a novel therapeutic target, and if salvageable, may alter the time course of WMH progression. Pathologically, WMH-P may correspond to mild tissue changes with slightly lower myelin density, activated endothelial cells, looser but still largely intact axonal networks, and normal glial density (Ding et al., 2023). Although these changes cannot be detected using conventional MRI scans, they have been shown to be associated with cognitive impairment on DTI/DKI scans (Brandhofe et al., 2021).

A cross-sectional study showing structural and blood flow changes in the brain tissue around the WMH by DTI/DKI and ASL demonstrated that WM injury extends beyond the range of visible lesions commonly seen on conventional MRI. The cerebral blood flow (CBF) penumbra was larger than the tectonic penumbra in the deep WMH penumbra (DWMH-P), but in the periventricular WMH penumbra (PVWMH-P), the CBF and tectonic penumbra were almost identical in extent. These findings suggest that CBF reduction may precede microstructural deterioration of NAWM tissue (Wu et al., 2019; Promjunyakul et al., 2016). However, it is worth noting that some longitudinal studies have found that changes in the penumbra microstructure correlate with progression, implying a new target for treatment (Jiaerken et al., 2019; van Leijsen et al., 2018). Changes in penumbral signaling may further our understanding of the underlying etiology of early WMH development and expansion. This is essential for preventing WMH growth and subsequent cognitive and motor deficits. At the same time, the discovery of early reversible changes will redefine the CSVD marker of WMH, which can be slowed or reversed by treatment (Wang et al., 2023). The latest DTI studies are not only limited to patients with CSVD who have already developed the disease but also aim to apply DTI data to identify the early clinical stage (VaMCI) or even the preclinical stage (NCI) of CSVD. A recent study showed that damage to the corpus callosum and internal and external capsules was detected in NCI, and the same results were observed in a previous study of VaMCI patients with CSVD (Papma et al., 2014). It was further found that the uncinate fasciculus of the frontal and temporal lobes, which are closely related to executive function, was impaired in the NCI group, and the MD of the cingulate gyrus (CGC) was increased in the NCI group compared to healthy controls. This suggests that impairment of cerebral WM integrity also occurs in the preclinical stage of vascular cognitive impairment (VCI) due to CSVD (Du et al., 2021).

## 6 Application of new technologies in CSVD research

Most current CSVD studies have been analyzed using DTI. Although DTI is significantly better than conventional MRI in determining nerve fiber myelin lesions due to its technical limitations, it is not effective in determining the integrity of nerve myelin in brain regions with low anisotropy or bias toward anisotropy, such as the arcuate fibers in the subcortical WM (Guo et al., 2016; Guo et al., 2016).

Diffusion kurtosis imaging (DKI) is an extension of the DTI technique that partially overcomes these limitations by more sensitive and accurate detection of alterations in the microstructure of nerve fiber bundles, which are particularly significant in the subcortex (Chen et al., 2017). DKI assumes that the dispersion of water molecules in living tissues is non-Gaussian (Deng et al., 2023). DKI was used to compensate for the lack of a second-order tensor by adding a fourth-order tensor correction term to the imaging equation, forming a convex surface with multiple spines to coincide with the multifiber orientation, thus characterizing the extent to which water molecule dispersion deviates from a normal distribution (Wei et al., 2021). It requires high b-values (typically b > 1,000 s/ mm2) as well as the application of diffusion-sensitive gradient fields in multiple (≥15) directions. In addition to the conventional diffusion parameters MD and FA, mean kurtosis (MK), radial kurtosis (RK), axial kurtosis (AK), and kurtosis anisotropy (KA) can be used (Qi et al., 2023). DKI can detect these microstructural changes even before any imaging findings are discovered through conventional imaging, which is why it is superior to DTI (Marrale et al., 2016). It has been shown that DKI has high sensitivity for the detection of subclinical brain damage in CSVD, in which MK is more sensitive in responding to the degree of brain function impairment (Tong et al., 2019). However, to date, the use of DKI to study CSVD is still in the minority and has mostly focused on the exploration of CSVD-related depression (Li et al., 2020) and cognitive functioning (Liu et al., 2021; Liu et al., 2023; Liu et al., 2019).

## 7 Discussion

CSVD has significant global influence (Markus and de Leeuw, 2023). Much of the recent research has focused on changes in the body fluid environment and MRI preclinical manifestations of CSVD, in addition to traditional studies of white matter bundles and structural networks. Because processes such as damage to the LAS (Zhang et al., 2023), disorders of the GS (Tian et al., 2023), and increased extracellular fluid volume (Lan et al., 2022) are now considered to be the key mechanisms of microstructural WM injury, this suggests that abnormalities in fiber organization may be secondary mechanisms (Duering et al., 2018; Muñoz Maniega et al., 2017). The discovery of WMH-P (Wang et al., 2023) similarly proves that the same destructive pathologic changes occur in the NAWM around the WMH. Identifying and evaluating such specific and subtle changes in the penumbra and the early development of therapeutic strategies based on etiology will be able to slow down or salvage the progression of WMH, which may be a new therapeutic goal.

In this review, we found that the development and improvement of DTI analysis methods by describing changes in the organizational properties of specific WM tracts (focusing on the observation of localized white matter fibers) to investigate changes in the topological properties between different brain regions (focusing on the observation of connections in different brain regions or even whole-brain connectivity) have continuously increased the breadth and depth of DTI applications. However, we found that due to the different clinical manifestations of CSVD, numerous disease-lesion mappings have been produced, both for WM bundle damage studies and for studies of brain network changes. Owing to the limitations of available therapeutic techniques, such articles, although providing in-depth investigations of pathogenic mechanisms, lack clinical guidance in some sense. Recent DTI studies have highlighted the relationship between disease onset and damage to the WM tract connectivity (i.e., the brain structural network) in the whole brain, which can be used to prevent the onset and further progression of CSVD by exploring the etiology of CSVD and intervening in a timely manner in the clinical setting or in life. Therefore, the potential mechanism of WM bundle damage and the microstructural pathological changes of NAWM in the preclinical stage are becoming new hot spots in CSVD research.

This shift in research direction is likewise in response to the need for clinical work, and there have been many DTI studies related to CSVD. However, the pathogenic mechanism of CSVD remains ambiguous and no consensus has been reached regarding the standardized treatment of CSVD. The ethical implications of advanced neuroimaging in CSVD are also a concern due to issues of access and data analysis of DTI technology, as well as the protection of patients’ rights and interests, with advanced neuroimaging technology, which is often costly and scarcely equipped. This may lead to unequal distribution of healthcare resources among patients in different regions and from different socioeconomic backgrounds and give rise to ethical controversies. Ensuring that all patients in need have access to advanced diagnostic technologies is an important ethical consideration. Neuroimaging data are often complex and difficult to understand intuitively and need to be interpreted by doctors or specialists with specialized knowledge and experience. This requires healthcare organizations to strengthen staff training and improve their interpretation skills. Neuroimaging data contains a large amount of sensitive personal information, and strict privacy protection regulations must be followed to ensure data security and anonymity. Together, these aspects form an important ethical framework for advanced neuroimaging for the diagnosis and treatment of CSVD.

Moreover, CSVD progresses insidiously, and patients often have no typical symptoms in the early stage; however, the risk of stroke, dementia, and death is increased, and the long-term prognosis is poor. Therefore, research on early diagnosis and pathogenesis of CSVD is particularly important. Follow-up studies could be carried out on the following aspects: (1) based on the current DTI study, more advanced DKI technology was used to study the WM microstructure damage mechanism of CSVD; (2) a prospective longitudinal multimodal, multicenter, large-sample study; and (3) improved algorithms for DTI/DKI using AI, deep learning, etc., to enable patients to be accurately screened at the preclinical stage of CSVD. In conclusion, future prospective studies should be patient oriented and focus on the etiology and preclinical diagnosis of CSVD to provide reliable imaging evidence to support clinical prevention and treatment.
